# Supplementary material for: Pyruvate Dehydrogenase Kinase 1 inhibition mediated oxidative phosphorylation enhancement in cartilage promotes osteoarthritis progression
Source: BMC Musculoskelet Disord. 2023 Jul 20;24:597. doi: 10.1186/s12891-023-06585-6 (PMC10357736; doi:10.1186/s12891-023-06585-6)
Supplement: Supplementary file 1 — Additional file 1. [file 12891_2023_6585_MOESM1_ESM.zip › BP.Gsea.1653623667859/GOBP_FIBROBLAST_APOPTOTIC_PROCESS.html]

Details for gene set GOBP\_FIBROBLAST\_APOPTOTIC\_PROCESS[GSEA]

|  || Dataset | 55457\_collapsed\_to\_symbols.cls.cls #OA\_versus\_normal.cls.cls #OA\_versus\_normal\_repos |
| Phenotype | cls.cls#OA\_versus\_normal\_repos |
| Upregulated in class | normal |
| GeneSet | GOBP\_FIBROBLAST\_APOPTOTIC\_PROCESS |
| Enrichment Score (ES) | -0.5632046 |
| Normalized Enrichment Score (NES) | -1.7214675 |
| Nominal p-value | 0.010791367 |
| FDR q-value | 0.12119248 |
| FWER p-Value | 1.0 |
Table: GSEA Results Summary

  

Fig 1: Enrichment plot: GOBP\_FIBROBLAST\_APOPTOTIC\_PROCESS      
 Profile of the Running ES Score & Positions of GeneSet Members on the Rank Ordered List

  

| SYMBOL | TITLE | RANK IN GENE LIST | RANK METRIC SCORE | RUNNING ES | CORE ENRICHMENT || 1 | GAS6 | growth arrest specific 6 [Source:HGNC Symbol;Acc:HGNC:4168] | 1942 | 0.368 | -0.0934 | No |
| 2 | SFRP1 | secreted frizzled related protein 1 [Source:HGNC Symbol;Acc:HGNC:10776] | 2503 | 0.303 | -0.0897 | No |
| 3 | CUL3 | cullin 3 [Source:HGNC Symbol;Acc:HGNC:2553] | 3480 | 0.204 | -0.1336 | No |
| 4 | CFDP1 | craniofacial development protein 1 [Source:HGNC Symbol;Acc:HGNC:1873] | 4464 | 0.116 | -0.1919 | No |
| 5 | TP53 | tumor protein p53 [Source:HGNC Symbol;Acc:HGNC:11998] | 5511 | 0.032 | -0.2682 | No |
| 6 | BID | BH3 interacting domain death agonist [Source:HGNC Symbol;Acc:HGNC:1050] | 5715 | 0.014 | -0.2817 | No |
| 7 | PRDM11 | PR/SET domain 11 [Source:HGNC Symbol;Acc:HGNC:13996] | 5876 | 0.004 | -0.2935 | No |
| 8 | TP63 | tumor protein p63 [Source:HGNC Symbol;Acc:HGNC:15979] | 6274 | -0.025 | -0.3204 | No |
| 9 | STK17A | serine/threonine kinase 17a [Source:HGNC Symbol;Acc:HGNC:11395] | 7016 | -0.081 | -0.3652 | No |
| 10 | PIK3CA | "phosphatidylinositol-4,5-bisphosphate 3-kinase catalytic subunit alpha [Source:HGNC Symbol;Acc:HGNC:8975]" | 7733 | -0.136 | -0.3996 | No |
| 11 | PIK3CG | "phosphatidylinositol-4,5-bisphosphate 3-kinase catalytic subunit gamma [Source:HGNC Symbol;Acc:HGNC:8978]" | 7953 | -0.152 | -0.3929 | No |
| 12 | BAK1 | BCL2 antagonist/killer 1 [Source:HGNC Symbol;Acc:HGNC:949] | 9266 | -0.253 | -0.4553 | No |
| 13 | STK17B | serine/threonine kinase 17b [Source:HGNC Symbol;Acc:HGNC:11396] | 9825 | -0.303 | -0.4513 | No |
| 14 | BTG1 | BTG anti-proliferation factor 1 [Source:HGNC Symbol;Acc:HGNC:1130] | 11267 | -0.466 | -0.4905 | Yes |
| 15 | CHD8 | chromodomain helicase DNA binding protein 8 [Source:HGNC Symbol;Acc:HGNC:20153] | 11825 | -0.554 | -0.4473 | Yes |
| 16 | BCL2L11 | BCL2 like 11 [Source:HGNC Symbol;Acc:HGNC:994] | 11867 | -0.564 | -0.3625 | Yes |
| 17 | API5 | apoptosis inhibitor 5 [Source:HGNC Symbol;Acc:HGNC:594] | 12275 | -0.670 | -0.2897 | Yes |
| 18 | MYC | "MYC proto-oncogene, bHLH transcription factor [Source:HGNC Symbol;Acc:HGNC:7553]" | 12801 | -0.970 | -0.1792 | Yes |
| 19 | XRCC2 | X-ray repair cross complementing 2 [Source:HGNC Symbol;Acc:HGNC:12829] | 12882 | -1.198 | 0.0014 | Yes |
Table: GSEA details [plain text format]

  

Fig 2: GOBP\_FIBROBLAST\_APOPTOTIC\_PROCESS      
 Blue-Pink O' Gram in the Space of the Analyzed GeneSet

  

Fig 3: GOBP\_FIBROBLAST\_APOPTOTIC\_PROCESS: Random ES distribution      
 Gene set null distribution of ES for **GOBP\_FIBROBLAST\_APOPTOTIC\_PROCESS**
